# Association between wanting circumcision and risky sexual behaviour in Zimbabwe: evidence from the 2010–11 Zimbabwe demographic and health survey

**DOI:** 10.1186/s12978-015-0001-3

**Published:** 2015-03-07

**Authors:** Antony Chikutsa, Alfred C Ncube, Shepard Mutsau

**Affiliations:** Zimbabwe Open University, Harare, Zimbabwe

**Keywords:** Male circumcision, Risky sexual behaviour, HIV prevention

## Abstract

**Background:**

Zimbabwe adopted voluntary medical male circumcision (VMMC) as an additional HIV prevention strategy in 2009. A number of studies have been conducted to understand the determinants of VMMC uptake but few studies have examined the characteristics of men who are willing to get circumcised or the link between wanting circumcision and risky sexual behaviour. This study investigated the relationship between wanting male circumcision and engaging in risky sex behaviours. This was based on the assumption that those who are willing to undergo circumcision are already engaging in risky sexual behaviours.

**Data and methods:**

Data from men age 15–45 years who were interviewed during the 2010–11 Zimbabwe Demographic and Health Survey of 2010–11 was used. A total of 7480 men were included in the sample for this study. Logistic regression was used to assess the association between wanting circumcision and risky sexual behaviours.

**Findings:**

Men in the highest wealth tercile were significantly more likely to want circumcision compared to men in lower wealth terciles (OR = 1.36, p < 0.01). Wanting circumcision was also significantly associated with age. Men in the 25–34 age category reported wanting circumcision more (OR = 1.21, p < 0.05) while older men were significantly less likely to want circumcision (OR = 0.63, p < 0.01). Christian men and those residing in rural areas were also less likely to want circumcision (OR = 0.74, p < 0.05 and OR = 0.75, p < 0.001 respectively). The findings of this study indicate a strong association between wanting circumcision and having had risky sex (OR = 1.36, p < 0.01), having multiple partners (OR = 1.35, p < 0.01) and having paid for sex (OR = 1.42, p < 0.001) However, wanting circumcision was negatively associated with having used a condom at the last risky sex (OR = 0.76, p < 0.001).

**Conclusions:**

The association between demand for VMMC and risky sexual behaviour need continuous monitoring. We emphasise that the promotion of VMMC for HIV prevention should not overshadow the promotion of existing methods of HIV prevention such as condoms and reduction of sexual partners.

## Introduction

Voluntary medical male circumcision (VMMC) has become an important aspect of HIV prevention strategies in Zimbabwe following its adoption in 2009 as an additional HIV prevention strategy. The role out campaign of VMMC follows findings that circumcision is efficacious in preventing HIV transmission among heterosexual men [1-3]. Zimbabwe has a low circumcision rate currently estimated at about 9.2 percent [4]. Given the low circumcision rate, previous studies have attempted to establish the acceptability of male circumcision as an HIV prevention strategy among men in Zimbabwe. Findings from earlier studies indicate strong acceptability of circumcision for HIV prevention in Zimbabwe [5,6]. Studies which investigated acceptability of circumcision in other countries also found support for the promotion of VMMC for HIV prevention [7,8]. Subsequent studies have shown a high level of knowledge of VMMC among women and men in Zimbabwe [6,9]. Studies on circumcision and behavioural disinhibition among circumcised men in Kenya, Malawi and Zimbabwe have also allayed fears that circumcised men engage in risky sexual behaviour [10-12]. However, in Uganda, it was found that circumcised men engaged in riskier sex behaviour compared to uncircumcised men although their HIV prevalence rate is lower [13]. Thus, public health campaigns to promote the uptake of VMMC have focussed on demand creation for circumcision. However, the response to these campaigns in Zimbabwe has not met the expected targets because less than 200,000 men have been circumcised against a target of 1.9 million for the period 2010–2015 [9].

The discourse on the uptake of VMMC for HIV prevention is ongoing. There is need to understand the characteristics of men who volunteer for male circumcision. This study intends to contribute to this discourse by evaluating whether men who want a circumcision are already engaging in risky sexual behaviour.

### Conceptual framework

According to the Cognitive Dissonance Theory, human beings become stressed when their behaviour is inconsistent with their cognitions (beliefs, values, ideas) and will strive to restore equilibrium through the avoidance of certain information/behaviour that is likely to increase the dissonance or through the adoption of behaviours that are in sync with their cognitions [14]. In the context of male circumcision for HIV prevention, men have information on how HIV is transmitted and they also know that their behaviour puts them at risk of getting infected. Studies in Zimbabwe have established that heterosexual contact accounts for 92 percent of all HIV infections [15] and that 82 percent of men recognise the risk factors associated with HIV infection such as inconsistent condom use and having multiple sex partners [4]. Thus, for men who know that their behaviour puts them at risk of getting infected with HIV, accepting male circumcision becomes a plausible remedy for the cognitive discomfort. It is therefore hypothesised that men who engage in risky sexual behaviours are likely to consider getting a circumcision for HIV prevention.

**Figure 1:**
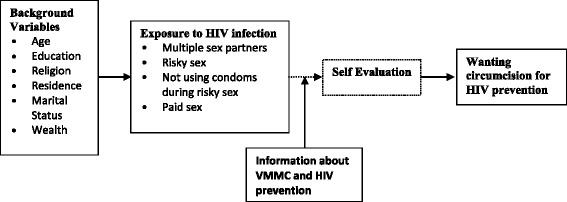
**Factors associated with wanting circumcision for HIV prevention.**

In coming up with the conceptual framework for this study (Figure 1), individual differences in exposure to HIV infection and hence to their willingness to adopt male circumcision were taken into account. Seven background variables were included in this analysis namely age, level of education, religion, place of residence (rural/urban), marital status, wealth status, and province of residence. Studies have found these background variables to be significantly associated with risky sexual behaviour among men [16-19]. Age, for example, determines whether one is sexually active or not while marital status is associated with exposure to unprotected sex [16]. Similarly, studies have shown that religion is significantly associated with HIV infection [16,20]. In the conceptual framework, background variables provide general guidelines on the pattern of sexual behaviour which will in turn influence exposure to HIV infection [16]. A person’s self evaluation of their sexual behaviour (in the third box) will lead them to conclude whether they need additional protection (male circumcision) from HIV or not.

## Data and methods

The methodology used in this study is similar to the one used in an earlier study which focused on the association between circumcision status and risky sexual behaviour [12]. The study relied on data on men between the ages of 15 and 54 years obtained from the Zimbabwe Demographic and Health Survey (ZDHS) of 2010–11. 7,480 men were interviewed in the survey on their circumcision status and sexual behaviour. Individual and household characteristics included in the analysis include age, level of education, religion, province and urban/rural residence. These background variables were considered in order to understand how willingness to get a circumcision varies across these factors. The categories of religion, education and province were regrouped with a view to reducing the number of categories in the usual ZDHS reports. Men who participated in the survey were asked if they were circumcised or not. Those who reported to be uncircumcised were asked if they were willing to undergo circumcision if the procedure was made available. Risky sexual behaviour was measured using four indicators as follows:

- *Multiple sexual partners:* multiple sex partners in this study were defined as having had two or more sex partners in the 12 months preceding the survey.
  *Risky sex:* Risky sex in the present study is defined as any sexual encounter with a non-marital or non cohabiting partner in the 12 months before the survey among men who had sex in that period.
- *Condom use during last risky sexual encounter:* Condom use during sexual intercourse is considered a preventive measure against the transmission of STIs. The ZDHS collected data on condom use during different sexual encounters. However, for this study we only considered condom use at the last risky sexual intercourse among men who reported having had risky sex in the 12 months prior to the survey.
- *Paid sex:* In the present study, all men who reported to have ever paid for sex were considered to have engaged in risky sexual behaviour.

Data was analysed in STATA using bivariate and multiple regression techniques. The analysis tested the strength of the association firstly between wanting circumcision and background variables such as age, place of residence, education, religion and marital status on one hand, and secondly, the association between wanting circumcision and engaging in risky sexual behaviours on the other. We applied the ZDHS weight variable so that the outcome of this analysis can reflect the complex multi-stage sampling designs that are usually employed in Demographic and Health Surveys. The indicators of risky sexual behaviour and the associated denominators are shown in Table 1.

**Table 1:** **Indicators of risky sexual behavior**

**Indicator**	**Weighted percent**	**Denominator**
Multiple sexual partners	10.5	7,480
Risky sex	33.2	5,100
Paid sex	18.0	7,480
Did not use condom at last risky sex^a^	22.3	1,686

^a^In eight weighted cases, men who had risky sex in the past 12 months were excluded because their last 3 partners were spouses; condom use with non-spousal partner could not be determined.

## Results

### Factors associated with wanting a circumcision

The ZDHS interviewed 7480 men and 9.2 percent of these reported to be circumcised while 90.8 percent were not circumcised. The 6795 men who are not circumcised were asked if they would consider getting circumcised if circumcision was made free and safe. Thirty six percent or 2552 men said they would consider getting circumcised. A further analysis of the factors associated with willingness to get a circumcision using logistic regression models showed that age was significantly associated with wanting circumcision. The odds of wanting circumcision decrease as age increases. After adjusting for other factors, men in the 25–34 years age group were significantly more likely to want circumcision compared to men in the reference category (OR = 1.21, p < 0.05) while those in the 45–54 years age category were significantly less likely to want a circumcision (OR = 0.63, p < 0.01). Also, men in Matabeleland/Midlands and Bulawayo were significantly more likely to want a circumcision than men in the other provinces (OR = 1.41, p < 0.01 and 1.73, p < 0.01 respectively). Similarly, married or formerly married men were significantly more likely to want a circumcision than men who have never married (OR = 1.34, p < 0.01 and 1.57, p < 0.01 respectively). Men in the highest wealth tercile were also significantly likely to report wanting circumcision compared to men in the first two terciles (OR = 1.36, p < 0.01). Level of education and rural/urban residence were not significantly associated with wanting circumcision after adjusting for other factors. The unadjusted and adjusted odds ratios of wanting a circumcision by background factors are presented in Table 2.

**Table 2:** **Factors associated with willingness to get a circumcision (n = 7480)**

**Variable**	**Unadjusted ORs**	**Adjusted ORs**
Age		
15-24	1.00	1.00
25-34	1.47*** [1.29-1.66]	1.21* [1.01-1.44]
35-44	1.31*** [1.13-1.51]	1.01 [0.82-1.24]
45-54	0.75** [0.61-0.93]	0.63** [0.48-0.84]
Level of education		
None	1.00	1.00
Primary	1.53 [0.87-2.68]	1.40 [0.78-2.51]
Secondary+	2.45** [1.40-4.28]	1.98 [1.10-3.56]
Religion		
Traditional/Other	1.00	1.00
Christians	0.79 [0.59-1.08]	0.74* [0.54-0.99]
Muslim^+^	2.30 [0.48-10.8]	1.70 [0.34-8.42]
None	0.68* [0.49-0.93]	0.67* [0.49-0.91]
Residence		
Urban	1.00	1.00
Rural	0.75*** [0.66-0.85]	1.02 [0.82-1.29]
Province		
Manicaland	1.00	1.00
Mashonaland	1.00 [0.79-1.27]	1.06 [0.84-1.32]
Matabeleland/Midlands	1.27* [1.00-1.61]	1.41** [1.12-1.76]
Harare	1.23 [0.94-1.61]	1.06 [0.77-1.44]
Bulawayo	1.96*** [1.45-2.65]	1.73** [1.23-2.42]
Marital status		
Never married	1.00	1.00
Married/Living together	1.26*** [1.13-1.40]	1.34** [1.13-1.59]
Formerly married	1.44** [1.14-1.82]	1.57** [1.19-2.07]
Wealth tercile		
Lowest	1.00	1.00
Middle	1.11 [0.95-1.28]	1.12 [0.97-1.29]
Highest	1.49*** [1.30-1.72]	1.36** [1.11-1.67]

Exponential coefficients; 95percent confidence intervals in brackets.

*p < 0.05, **p < 0.01, ***p < 0.001.

^+^Based on few weighted cases.

“Secondary+” includes those with a secondary education and above.

“Mashonaland Province” includes Mashonaland East, Mashonaland West, Mashonaland Central and Masvingo.

“Matabeleland/Midlands” includes Matabeleland North, Matabeleland South and Midlands.

### Wanting a circumcision and risky sexual behaviour

We also assessed a possible link between wanting a circumcision and engaging in risky sexual behaviour. Four measures of risky sexual behaviour were considered as outlined earlier. We used logistic regression models to compute unadjusted and adjusted odds ratios and the results are shown in Tables 3,4, 5, 6. The results show that wanting a circumcision is significantly associated with all the four proxies of risky sexual behaviour even after adjusting for background variables. Wanting circumcision was significantly associated with having ever paid for sex (OR = 1.42, p < 0.001). The odds of having ever paid for sex increase with age and are also higher among married and formerly married men (p < 0.001). Men in the middle wealth tercile had significantly higher odds of having ever paid for sex compared to men in other wealth categories. The odds of having ever paid for sex were significantly lower among Christian men (OR = 0.54, p < 0.001), among men living in rural areas (OR = 0.55, p < 0.001), among men in Matabeleland/Midlands (OR = 0.74, p < 0.05) and Bulawayo (OR = 0.31, p < 0.001).

**Table 3:** **Unadjusted and adjusted odds ratios of wanting a circumcision and having ever paid for sex (n = 7480)**

**Variable**	**Unadjusted ORs**	**Adjusted ORs**
Wanting a circumcision	1.40*** [1.22-1.62]	1.42*** [1.22-1.66]
Age		
15-24	1.00	1.00
25-34	4.58*** [3.69-5.69]	2.47 *** [1.81-3.35]
35-44	6.10*** [4.91-7.58]	3.11*** [2.20-4.39]
45-54	10.3*** [7.95-13.3]	5.80*** [3.99-8.44]
Level of education		
None	1.00	1.00
Primary	1.29 [0.53-3.09]	1.46 [0.55-3.86]
Secondary+	1.08 [0.45-2.56]	1.40 [0.52-3.71]
Religion		
Traditional/Other	1.00	1.00
Christians	0.37*** [0.27-0.49]	0.54*** [0.38-0.74]
Muslim^+^	0.86 [0.43-1.69]	1.86 [0.49-7.01]
None	0.68* [0.48-0.93]	0.98 [0.70-1.35]
Residence		
Urban	1.00	1.00
Rural	0.69*** [0.58-0.80]	0.55*** [0.41-0.76]
Province		
Manicaland	1.00	1.00
Mashonaland	0.88 [0.69-1.12]	0.83 [0.63-1.09]
Matabeleland/Midlands	0.79 [0.62-1.02]	0.74* [0.55-0.99]
Harare	1.29 [0.98-1.69]	0.81 [0.56-1.18]
Bulawayo	0.53** [0.35-0.79]	0.31*** [0.18-0.50]
Marital status		
Never married	1.00	1.00
Married/Living together	5.19*** [4.33-6.23]	2.13*** [1.58-2.87]
Formerly married	7.70*** [5.7-10.3]	3.39*** [2.30-4.98]
Wealth tercile		
Lowest	1.00	1.00
Middle	1.16 [0.94-1.43]	1.25* [1.03-1.53]
Highest	1.36*** [1.14-1.61]	1.16 [0.85-1.58]

Exponential coefficients; 95percent confidence intervals in brackets.

*p < 0.05, **p < 0.01, ***p < 0.001.

^+^Based on few weighted cases.

“Secondary+” includes those with a secondary education and above.

“Mashonaland Province” includes Mashonaland East, Mashonaland West, Mashonaland Central and Masvingo.

“Matabeleland/Midlands” includes Matabeleland North, Matabeleland South and Midlands.

**Table 4:** **Unadjusted and adjusted odds ratios of wanting a circumcision and condom use at last risky sex (n = 1686)**

**Variable**	**Unadjusted ORs**	**Adjusted ORs**
Wanting a circumcision	0.67** [0.52-0.87]	0.76* [0.57-0.99]
Age		
15-24	1.00	1.00
25-34	0.62*** [0.46-0.83]	0.67* [0.47-0.98]
35-44	0.71 [0.47-1.08]	0.74 [0.42-1.29]
45-54	0.75 [0.39-1.43]	0.59 [0.27-1.28]
Level of education		
None	1.00	1.00
Primary	0.75 [0.17-3.32]	1.28 [0.19-8.51]
Secondary+	0.33 [0.07-1.50]	0.70 [0.09-4.91]
Religion		
Traditional/Other	1.00	1.00
Christian	1.57 [0.77-3.20]	1.46 [0.67-3.19]
Muslim^+^	1.00	1.00
None	1.75 [0.83-3.67]	1.38 [0.63-3.03]
Residence		
Urban	1.00	1.00
Rural	1.61*** [1.23-2.11]	0.85 [0.48-1.51]
Province		
Manicaland	1.00	1.00
Mashonaland	1.31 [0.80-2.17]	1.25 [0.71-2.19]
Matabeleland/Midlands	2.05** [1.24-3.39]	1.86* [1.04-3.32]
Harare	0.86 [0.49-1.47]	1.01 [0.46-2.23]
Bulawayo	0.99 [0.54-1.84]	1.06 [0.43-2.57]
Marital status		
Never married	1.00	1.00
Married/Living together	0.78 [0.57-1.07]	1.01 [0.65-1.54]
Formerly married	0.79 [0.53-1.17]	0.88 [0.52-1.48]
Wealth tercile		
Lowest	1.00	1.00
Middle	0.69* [0.51-0.93]	0.86 [0.61-1.23]
Highest	0.42*** [0.31-0.57]	0.61* [0.38-0.98]

Exponential coefficients; 95percent confidence intervals in brackets.

*p < 0.05, **p < 0.01, ***p < 0.001.

^+^Based on few weighted cases.

“Secondary+” includes those with a secondary education and above.

“Mashonaland Province” includes Mashonaland East, Mashonaland West, Mashonaland Central and Masvingo.

“Matabeleland/Midlands” includes Matabeleland North, Matabeleland South and Midlands.

**Table 5:** **Unadjusted and adjusted odds ratios of wanting a circumcision and having multiple sex partners (n = 7480)**

**Variable**	**Unadjusted ORs**	**Adjusted ORs**
Wanting a circumcision	1.44*** [1.20-1.73]	1.35** [1.12-1.63]
Age		
15-24	1.00	1.00
25-34	2.03*** [1.67-2.46]	0.87 [0.62-1.23]
35-44	1.52** [1.19-1.94]	0.61** [0.42-0.88]
45-54	1.27 [0.91-1.76]	0.61** [0.41-0.91]
Level of education		
None	1.00	1.00
Primary	0.95 [0.37-2.45]	0.95 [0.33-2.65]
Secondary+	1.10 [0.43-2.85]	1.18 [0.43-3.21]
Religion		
Traditional/Other	1.00	1.00
Christians	0.61* [0.41-0.91]	0.59** [0.40-0.88]
Muslim^+^	0.94 [0.27-3.28]	0.44 [0.04-5.01]
None	0.97 [0.65-1.42]	0.97 [0.66-1.43]
Residence		
Urban	1.00	1.00
Rural	0.84 [0.68-1.03]	0.70 [0.47-1.03]
Province		
Manicaland	1.00	1.00
Mashonaland	0.76 [0.56-1.05]	0.69* [0.50-0.96]
Matabeleland/Midlands	0.81 [0.58-1.13]	0.75 [0.53-1.06]
Harare	0.97 [0.66-1.42]	0.81 [0.51-1.30]
Bulawayo	0.66* [0.44-0.99]	0.59* [0.35-0.98]
Marital status		
Never married	1.00	1.00
Married/Living together	2.36*** [1.93-2.90]	2.99*** [2.10-4.30]
Formerly married	2.42*** [1.59-3.66]	2.94*** [1.66-5.21]
Wealth tercile		
Lowest	1.00	1.00
Middle	0.92 [0.71-1.19]	0.88 [0.69-1.11]
Highest	0.95 [0.76-1.72]	0.79 [0.55-1.12]

Exponential coefficients; 95percent confidence intervals in brackets.

*p < 0.05, **p < 0.01, ***p < 0.001.

^+^Based on few weighted cases.

“Secondary+” includes those with a secondary education and above.

“Mashonaland Province” includes Mashonaland East, Mashonaland West, Mashonaland Central and Masvingo.

“Matabeleland/Midlands” includes Matabeleland North, Matabeleland South and Midlands.

**Table 6:** **Unadjusted and adjusted odds ratios of wanting a circumcision and risky sex (n = 5100)**

**Variable**	**Unadjusted ORs**	**Adjusted ORs**
Wanting a circumcision	1.23** [1.08-1.41]	1.36** [1.06-1.74]
Age		
15-24	1.00	1.00
25-34	0.15*** [0.12-0.18]	0.52*** [0.37-0.72]
35-44	0.06*** [0.05-0.08]	0.35*** [0.25-0.49]
45-54	0.04*** [0.03-0.05]	0.24*** [0.15-0.37]
Level of education		
None	1.00	1.00
Primary	2.95** [1.44-6.07]	3.79 [0.39-36.1]
Secondary+	3.64*** [1.77-7.48]	4.14 [0.44-38.8]
Religion		
Traditional/Other	1.00	1.00
Christians	1.36 [0.97-1.92]	0.51** [0.31-0.82]
Muslim^+^	1.23 [0.49-3.06]	0.05*** [0.02-0.15]
None	1.76 [1.24-2.49]	0.86 [0.54-1.39]
Residence		
Urban	1.00	1.00
Rural	0.78** [0.67-0.90]	0.66** [0.44-0.99]
Province		
Manicaland	1.00	1.00
Mashonaland	0.82 [0.63-1.08]	0.88 [0.57-1.35]
Matabeleland/Midlands	1.41* [1.06-1.88]	1.18 [0.74-1.85]
Harare	1.24 [0.92-1.67]	1.21 [0.74-1.99]
Bulawayo	1.69** [1.25-2.29]	0.83 [0.49-1.39]
Marital status		
Never married	1.00	1.00
Married/Living together	0.00*** [0.00-0.00]	0.00*** [0.00-0.00]
Formerly married	0.01*** [0.00-0.03]	0.02*** [0.00-0.05]
Wealth tercile		
Lowest	1.00	1.00
Middle	1.41*** [1.18-1.69]	0.95 [0.69-1.31]
Highest	1.43*** [1.21-1.70]	0.96 [0.64-1.44]

Exponential coefficients; 95percent confidence intervals in brackets.

*p < 0.05, **p < 0.01, ***p < 0.001.

^+^Based on few weighted cases.

“Secondary+” includes those with a secondary education and above.

“Mashonaland Province” includes Mashonaland East, Mashonaland West, Mashonaland Central and Masvingo.

“Matabeleland/Midlands” includes Matabeleland North, Matabeleland South and Midlands.

Condom use in the last risky sexual encounter was negatively associated with wanting male circumcision (OR = 0.76, p < 0.05). Condom use in the last risky sex was significantly lower among men in the 25–34 years age group (OR = 0.67, p < 0.05), and among men in the highest wealth tercile (OR = 0.61, p < 0.05). However, condom use was significantly higher among men in Matabeleland/Midlands (OR = 1.86, p < 0.05).

Having multiple sex partners in the 12 months preceding the survey was found to be significantly associated with wanting a circumcision (OR = 1.35, p < 0.01). The findings suggest that the odds of having multiple sex partners decrease with age (OR = 0.61, p < 0.01 for age groups 35–44 and 45–54 years). The odds of having multiple sex partners are significantly lower among Christian men (OR = 0.59, p < 0.01), among men in Matabeleland/Midlands (OR = 0.69, p < 0.05) and in Bulawayo (OR = 0.59, p < 0.05). However, the odds were significantly higher among married men (OR = 2.99, p < 0.001) and formerly married men (OR = 2.94, p < 0.001).

Wanting a circumcision was also found to be significantly associated with having had risky sex (OR = 1.36, p < 0.01). The odds of having risky sex decrease with age and were significantly lower among Christian and Muslim men (OR = 0.51, p < 0.01 and 0.05, p < 0.001 respectively), among men residing in rural areas (OR = 0.66, p < 0.01), and among married and formerly married men (p < 0.001).

## Discussion and conclusion

The promotion of male circumcision for HIV prevention prompted studies which evaluated support for the procedure particularly in areas where circumcision is not traditionally practiced. In Zimbabwe, such studies found a general support for the procedure [5,6]. Besides acceptability of the procedure, other studies investigated the possibility of behavioural disinhibition among circumcised men and found that circumcision is not significantly associated with risk compensation [10,11,21]. The study of the characteristics of men who are in need of circumcision is fairly recent. This study investigated the characteristics of men who are willing to undergo circumcision for HIV prevention. In particular, the study tested the hypothesis that wanting circumcision is associated with engaging in risky sexual behaviours among men who participated in the 2010–11 ZDHS. On the whole, the findings of this study support the hypothesis that wanting circumcision is significantly associated with engaging in risky sexual behaviour. This finding is similar to that obtained in Botswana [22].

The findings of this study show a significant association between age and wanting circumcision. Men in the 25–34 years age category were more likely than men in other age groups to want circumcision. Wanting circumcision was lowest among older men in the 45–54 years age group. An analysis of risky sexual behaviours showed that men in the 25–34 years category were least likely to have used a condom during the last risky sexual encounter and had higher odds of having had risky sex compared to men in other age groups. On the other hand, men in the 45–54 years age group had significantly lower odds of wanting circumcision for HIV prevention. These men also had lower odds of having had risky sex or having multiple sex partners compared to men in other age groups. It is therefore possible that men’s perception of risk of contracting HIV play a significant role in wanting circumcision. Moreover, the finding that wanting male circumcision is associated with age points to the need for age-specific programming during VMMC promotion campaigns. It has been suggested, for example, that older men need to be encouraged to participate in VMMC programmes so that they act as role models for younger men [15].

Religion is a significant determinant of male circumcision [23,24]. Studies, particularly among Muslim men, have suggested that religious doctrines on sexual relations play a part in controlling the extent to which followers of such doctrines are prone to engage in risky sexual behaviours [16,20]. The findings of this study showed that Christian men had significantly lower odds of having ever paid for sex, of having had multiple partners or having engaged in risky sex. The findings also show that Christian men were least likely to want a circumcision compared to those who reported to follow traditional religion. It is possible, again, that these men view their risk of getting infected with HIV as ‘low’ thus diminishing their need for circumcision. This finding has significant implications on the roll-out campaign considering that more than 70 percent of men who took part in the study reported to belong to a Christian grouping [4]. There is therefore a need to strengthen the involvement of Christian organisations in the roll-out campaign.

Married men or formerly married men had significantly higher odds of wanting circumcision compared to never married men. Married and formerly married men had higher odds of having ever paid for sex and of having multiple sex partners. It has been suggested that marriage exposes a person to frequent unprotected sex, supposedly with their spouse [16]. However, it is not clear why married men would have significant odds of having paid for sex or having multiple sex partners. What is clear though is that there could be a link between married/formerly married men’s perception of risk and them wanting circumcision as an additional method of HIV prevention.

The results of this study indicate that men in Matabeleland, Midlands and Bulawayo are more likely to want circumcision compared to men in other Provinces. While it has been suggested earlier that men’s perception of risk of getting infected with HIV seems to play a role in men’s willingness to get a circumcision, it is not clear whether the same explanation can be proffered for the willingness to get a circumcision among men in Matabeleland/Midlands and Bulawayo. These men had significantly lower odds of having ever paid for sex and significantly higher odds of having used a condom during the last risky sex. It is possible that the demand for circumcision in these Provinces could be a reflection of the cultural values of the people whose ancestors migrated from circumcising ethnic groups in South Africa. It is therefore recommended that these Provinces be prioritised in the allocation of resources because of the already existing demand for VMMC services. On the other hand, there is need for aggressive marketing of VMMC in the Mashonaland Provinces (and in rural areas) to stimulate demand. There are reports that programmes to encourage men to get circumcised are already underway [9].

In summary, men who want circumcision are likely to be young between the ages of 25–44 years, reside in Matabeleland/Midlands and Bulawayo Provinces, are married or formerly married and belong to the highest wealth tercile. In terms of risky sexual behaviours, men who want circumcision are likely to have not used a condom during the last risky sex and have higher odds of having had risky sex, of having multiple sex partners and of having ever paid for sex. The men least interested in getting a circumcision are older (in the 45–54 years age group), have never married, reside in the Mashonaland and Manicaland Provinces, and are Christians. In other words, men who want circumcision are more likely to be engaging in risky sexual behaviour. It is possible that this desire for circumcision from these men could be an outcome of the recent promotion of VMMC for HIV prevention. The campaigns could possibly have tilted men’s risk perception thus leading them to want ‘protection’ from male circumcision. However, there is need to continue emphasising the existing HIV prevention methods such as a reduction in the number of sexual partners and consistent condom use alongside VMMC.
